# The relationship between childhood SES and health in middle and old age: evidence from China

**DOI:** 10.3389/fpubh.2024.1396420

**Published:** 2024-10-15

**Authors:** Chen Yu-cheng, Gao Gong-jing

**Affiliations:** School of Political Science and Law, University of Jinan, Jinan, China

**Keywords:** childhood socio-economic status, health in middle and old age, life course, Propensity Score Matching, China

## Abstract

**Objective:**

Drawing from the extended Grossman health capital and demand theory and the life course theory, this study examined whether childhood SES has direct and significant correlation with health in middle and old age in a specific historical context in China.

**Methods:**

A sample of 9,861 respondents was obtained from the China Health and Retirement Longitudinal Study (CHARLS). Childhood SES was measured by objective indices of recall. Health was assessed by self-reported, physician diagnosis and the Center for Epidemiologic Studies Depression Scale (CESD). The Propensity Score Matching (PSM) was used to estimate the treatment effect between childhood SES and later life health. The Karlson-Holm-Breen (KHB) method was employed to examine the associative mediation effects.

**Results:**

Compared to respondents with low SES in childhood, respondents with high SES in childhood had, on average, 5.1% more likely to report their health as good, an average 2.4% lower prevalence of chronic diseases and an average 7.6% lower in the score of depression in middle and old age. The indirect relationships of childhood health, adulthood SES and adulthood lifestyle with health in middle and old age were all significant. SES upward mobility in adulthood can diminish the association between childhood disadvantage and poor health in middle and old age.

**Conclusion:**

The health effects of childhood SES can persist into middle and old age, this is more noticeable in rural areas, particularly in females. The critical period, cumulative risk and social mobility models produce synergistic effects in China. Our results also promote a paradigm shift in health interventions from old age to early life for health-vulnerable populations.

## 1 Introduction

China is aging rapidly, the health of the older adult is of great concern. Currently, the overall health status of older adult people is not optimistic in China. According to statistics, two-week prevalence and chronic diseases prevalence among individuals aged 65 and above has increased by 25 and 33% since 2008, respectively. The older adult have emerged as a prominent group suffering from chronic diseases. Simultaneously, there is a notable inclination of disease prevalence toward younger age groups. In 2023, the prevalence of chronic diseases among individuals under 60 reached 50 million. Chronic diseases not only affect an individual’s life quality, but also increase their medical and care burden because of the long duration, difficulty in curing and cumulative cost burden of it. Against this backdrop, how to maintain and improve the health of middle-aged and older adult individuals and extending healthy life expectancy in old age has become an urgent issue in the government agenda. In this study, we aim to explore the roots of healthy aging by examining whether childhood SES has direct and significant effect on health in middle and old age, as well as analyzing the mechanisms from a life course perspective under a specific historical context in China.

The factors affecting human health are numerous and complex, including lifestyle, genetic, environmental, medical and individual characteristics (1–4). In a disease spectrum dominated by chronic diseases, socioeconomic factors are increasingly associated with health. Health disparities resulting from SES have been existed since the industrial revolution. A longitudinal survey of British showed that the life expectancy of children from Duke families is approximately 20 years longer than their peers in other families in 1900 (5). Another longitudinal survey conducted in France between 1820 and 1870 also revealed that there exist significant disparities in life expectancy and mortality between high and low-income groups (6). A series of follow-up studies have similarly confirmed that individuals’ education, occupation, and income were significantly associated with objective (e.g., chronic, height) and subjective health indices (i.e., cognition, depression) (7–12).

Health is also a life course phenomenon, the current health status is also the product of a series of health decisions and inputs in early life (13, 14). Scholars began to investigate the before-and-after associations of health from a life course perspective since the 1960s (15). As childhood is a critical period for individual growth and development, studies from a micro-family perspective showed that father’s occupation and household income during an individual’s childhood have significant effects on their mortality, oral diseases, cognition and depression in adulthood (16–22). Studies from a macro-social perspective similarly indicated that experiencing major social events in early life is significantly associated with health in adulthood (23, 24). Beyond micro-family and macro-social factors, there exist well-established studies further explored the interactive effects of individual (e.g., SES) and environmental factors (e.g., basic infrastructure, formal care facilities and voluntary organizations) on health over time (25, 26). Therefore, the temporal correlates of health over the life course provides a valuable foundation for tracing the roots of “active ageing” (27).

The current study proposed several models explaining the cross-lifecycle effects of individual resources on health. The critical period model posited that SES at certain life stages has direct effects on later-life health and immune to later factors over time (28). In contrast, the cumulative risk model assumes that risk factors at different life stages accumulate over time and lead to negative effect on later-life health (29). The social mobility model indicated that upward mobility of SES in adulthood may eliminate or reduce the adverse health effects of early life disadvantage (30).

Studies on this subject have provided important implications for this paper. However, existing studies still have the following limitations: First, there is still much space for further exploration regarding the mechanism and group heterogeneity (e.g., gender, household registration) of the long-term health effect of childhood SES in China. Second, findings from foreign countries may not be applicable to Chinese society because of the unique historical context in China. For example, income could not reflect an individual’s SES because materials were distributed according to a plan during the planned economy era in China. While urban household registration is also viewed as high SES in the longer term in China. Third, most studies primarily focus on the older adult individuals, neglecting the middle-aged population despite the disease incidence is significantly younger.

Based on the extended Grossman health capital and demand theory and the life course theory, this study aims to examine whether childhood SES has direct and significant relationship with health in middle and old age in a specific historical context in China. This study also explored the specific mediating factors between childhood SES and health in later life and tested whether the critical period, cumulative risk and social mobility models produce synergistic effects in China. What’s more, we further analyzed the heterogeneity of the health effects of childhood SES in terms of household registration and gender.

## 2 Theoretical analysis

Different from the relationship between SES and health during the same period, the relationship between childhood SES and later-life health is superimposed on the former and the mechanisms are more complex. On the one hand, childhood SES depends on family rather than on themselves, such as parental SES and the family’s material living conditions. On the other hand, the intertemporal nature of health dictates the requirement for a life course perspective on the effect of childhood SES.

Jacobson (31) extended the Grossman model by further including household members as producers of individual health in the health production function. According to the extended Grossman health capital and demand model, parental SES is transmitted to minor children through a network of shared relationships within the family (20, 32, 33). Specifically, the production function of children’s health is subject to time and budget constraints. Parents with high SES have generally attained high education levels, they tend to prioritize the promotion of health literacy among their children. Consequently, good health literacy can increase children’s health output per unit of time. Meanwhile, parents with high SES generally earn higher incomes, which allows them to provide their children with more material resources necessary for mitigating health risks and improving health. As a result, high parental incomes and better material living conditions loosen the budget constraint within the health production function for children. In addition, life course theory emphasized that health is the product of a series of past health decisions and inputs (34). The critical period model also indicated that SES in childhood have unique and irreversible effects on health in later-life. *According to the theoretical analysis, we hypothesized that individuals with high SES in childhood would have a higher health level in middle and old age (Hypothesis 1).*

In terms of mechanisms, childhood health plays a mediating role. Differences in childhood SES expose the health production function of individuals to various time and budget constraints, which directly affect childhood health (35). Furthermore, individual health is a continuous state during the evolution of life and there is a correlation between health status at different life stages (27, 33, 36, 37). As a result, childhood health also become a critical factor in the health production function in adulthood, which in turn affects health in middle and old age (13). *We expected that high SES in childhood would improve individual’s childhood health and has a positive correlation with health in middle and old age (Hypothesis 2)*.

Adulthood SES plays a mediating role. Parents with high SES tend to prioritize their children’s education. As an investment in human capital, increasing educational level can help children acquire skills and qualifications needed for higher-paying careers in the future, and ultimately, raising their adulthood SES. In addition, children from families with high SES would typically benefit from intergenerational transfers of rich material resources when they reach adulthood (38, 39). High SES in adulthood indicates that individuals have stronger health investments and health risk resilience, which in turn have a positive effect on health in middle and old age (35). We hypothesized that *high childhood SES would improve individual’s SES in adulthood and has a positive correlation with health in middle and old age (Hypothesis 3)*.

Lifestyle plays a mediating role. Parents with high SES are more likely to avoid smoking, alcohol abuse and other unhealthy lifestyles in their daily lives because of their rich health knowledge and good health literacy. Parents’ healthy lifestyles will be passed on to their children due to the peer effect of household members, which in turn shapes the lifestyle of the children (40, 41). It is worth noting that once a healthy lifestyle is established, it will remain stable throughout the life cycle and thus improving health in middle and old age (42). We expected that *high SES in childhood would shape individual’s healthy lifestyles in adulthood, which would have a positive correlation with health in middle and old age (Hypothesis 4)*.

## 3 Methods

### 3.1 Data

The dataset using in this study is from the China Health and Retirement Longitudinal Survey (CHARLS) in 2018 and its 2014 Life Course Survey. CHARLS used a multi-stage stratified sampling method and covered 150 districts and counties in 28 provinces across the country. The respondents of CHARLS were people aged 45 years and above. The 2018 survey collected rich information on individuals’ health status, demographic characteristics, and household structure. The 2014 life course survey collected information on early life SES, living environment and parents’ health status during the respondents’ childhood. In this study, 17,229 individuals who were aged 45 and above at the 2018 survey and provided valid data from the 2014 survey. After excluding individuals with missing data on variables, 9,864 respondents were retained for the analytical model.

#### 3.1.1 Dependent variables

This study uses self-reported health to measure the comprehensive health status. Self-reported health is widely used in the previous studies and has been verified to accurately predict mortality, morbidity, and physician health ratings (43). CHARLS asked respondents how they assessed their health. With reference to previous literature, we assigned value 1 to those who answer “very good, good or fair.” Those who answered “not good or very bad” are assigned value 0 (44). To explore the effect of childhood SES on different dimensions of health, we included both objective and subjective health indices which were measured by chronic diseases prevalence and depress, respectively. The prevalence of chronic diseases is a dummy variable measured by whether the respondent had at least 1 of the 14 chronic diseases listed in the question (1 = yes, 0 = no). Depression was obtained based on the Center for Epidemiologic Studies Depression Scale (CESD). The CESD scale sums to a score range of 1–40. The higher the score, the more pronounced the depression (45).

#### 3.1.2 Independent variable

The independent variable in this study is the childhood SES. Childhood SES is a complicated concept without a standard measure or proxy in the existing literature. Generally, childhood SES is usually measured by parents’ income, education, and occupation type. In accordance with the common practices in the existing literatures and the realities of Chinses society, this study defines respondents’ childhood as 0–17 years old. We finally selected six indices to measure childhood SES, including parental education level (1 = educated, 0 = non-educated), parental occupation type (1 = non-agricultural occupations, 0 = farming), household registration (1 = urban, 0 = rural), household cooking fuel (1 = coal, electricity or natural gas; 0 = others), household drinking water (1 = tap water, 0 = others), and household energization (1 = yes, 0 = no). To facilitate analysis, we employed principal component analysis (PCA) to combine the six childhood SES indices into a composite childhood SES score. We considered childhood SES composite scores above the mean as high childhood SES and vice versa. In addition, this study also performed the robustness tests by replacing the childhood SES composite score with whether respondents experienced starvation in childhood.

#### 3.1.3 Mediating variables

According the theoretical analysis in the second part of this study, the mediating variables comprise childhood health, adulthood SES and adulthood lifestyle. Childhood health is measured by childhood self-reported health and bedridden due to illness. Adulthood SES is measured by a series of proxy variables such as current schooling years, occupation type, average monthly household expenditures, and household registration. Adulthood lifestyles are concerned with respondents’ smoking, drinking and physical exercising status.

#### 3.1.4 Control variables

In addition to childhood SES, respondents’ health is also influenced by demographic characteristics, household status and health endowment. Demographic characteristics include age, gender, and marriage status, while household status refer to number of siblings and children. As measuring health endowment directly is challenging, we utilize the health status of respondents’ parents as a proxy variable. Health endowment includes whether the respondent’s biological father or mother suffered from long-term bedridden, serious physical disabilities, insane or premature death during the respondent’s childhood. To estimate the health effect of childhood SES, we control for respondents’ demographic characteristics, household status and health endowment in the model.

### 3.2 Model

This study employs the Propensity Score Matching (PSM) method to estimate the effect of childhood SES on the health of middle-aged and older adult individuals. Compared to ordinary multiple regression models, PSM does not depend on the specific function form, which effectively reduces estimation bias caused by model function setting bias. PSM was proposed by Rosenbaum and Rubin in 1983. The core idea of PSM is to divide the sample into treatment and control groups based on categorical variables. Afterward, samples in the treatment and control groups with similar observable variables are matched using propensity scores. In this case, the matched control groups can be used as a counterfactual state of the treatment group (46). In this study, the high SES sample in childhood was designated as the treatment group, while the control group comprised the opposite.

The specific estimation process of the PSM method is as follows: First, a logit model is used to estimate the propensity score (the conditional probability of a respondent entering the treatment group); Second, different matching methods are employed to match treatment and control groups based on their propensity scores, ensuring that they are as similar as possible on a series of control variables. At this point, we can regard the differences in health between the treatment and control groups as an average treatment effect (ATT). The estimating equation is as follows:


(1) $$\begin{array}{l} ATT_{PSM}=E_{p\left(x_{i}\right)D_{i}|=1}\\ \left\{E\left[Y_{i}\left(1\right)|D_{i}=1,P\left(X_{i}\right)\right]-E\left[Y_{i}\left(0\right)|D_{i}=0,P\left(X_{i}\right)\right]\right\} \end{array}$$

In Equation 1, $ATT_{PSM}$ is the average treatment effect, $D_{i}$ is a dummy variable for high or low SES in childhood. $X_{i}$ is the control variable. $P\left(X_{i}\right)$ denotes the conditional probability of a respondent entering the treatment group. $E\left[Y_{i}\left(1\right)\right]$ and $E\left[Y_{i}\left(0\right)\right]$ denote the health of middle-aged and older adult individuals with high SES in childhood after matching. $E\left[Y_{i}\left(0\right)\right]$ is the health of middle-aged and older adult individuals with low SES in childhood after matching.

We also employ a decomposition method proposed by Breen et al. (47) (the KHB method) to decomposes the associative mediation effects of mediating variables. Compared to the traditional stepwise regression method for analyzing associative mediation effects, the KHB method is not restricted by the model specification. Furthermore, the KHB method can effectively incorporate multiple sets of mediating variables simultaneously, and estimate both the direct relationship of the independent variables with the dependent variable and the indirect relationship that result from the medicating variables (48).

## 4 Results

### 4.1 Descriptive analysis

The descriptive statistics in Table 1 shows that the majority of respondents reported good self-rated health and low CESD scores. However, an overwhelming percentage of participants confirmed the diagnosis of a chronic disease. In terms of childhood SES, most respondents had low SES in childhood. Only 30.9% of respondents had a composite score for childhood SES that exceeded average levels. Specifically, most respondents had rural household registration and their parents were uneducated and engaged in agricultural work during their childhood. Similarly, less than 50% of respondents experienced poor material living conditions during their childhood. Regarding control variables, the respondents had an average age of 60.8, with 49.2% being male. In addition, 88% of respondents were married or cohabiting, while over 97% reported having medical insurance. Respondents have more siblings than children. The overall health endowment of respondents is better. The proportion of parents who experienced long-term bedridden, serious physical disability or insanity was low in comparison to the proportion of parents who died young when the respondents were children.

**Table 1:** Descriptive statistics.

Variables	Mean	SD	Variables	Mean	SD
Dependent variables			Number of children	2.725	1.517
Self-reported health	0.747	0.435	Medical insurance	0.975	0.157
Chronic diseases	0.796	0.403	Biological mother died prematurely	0.281	0.449
CESD	18.150	6.482	Mother was sick in bed for a long-time during respondent’s childhood	0.124	0.330
Independent variable			Mother had severe physical disabilities during respondent’s childhood	0.026	0.159
Childhood SES	0.309	0.462	Mother was mentally ill during respondents’ childhood	0.027	0.161
Father’s education level	0.474	0.499	Biological father died prematurely	0.253	0.434
Mother’s education level	0.159	0.366	Father was sick in bed for a long-time during respondent’s childhood	0.082	0.274
Father’s occupation type	0.191	0.393	Father had severe physical disabilities during respondent’s childhood	0.028	0.164
Mother’s occupation type	0.070	0.255	Father was mentally ill during respondents’ childhood	0.014	0.120
Childhood household registration	0.074	0.255	Mediating variables		
Childhood household cooking fuel	0.232	0.422	Childhood self-reported health	0.875	0.331
Childhood drinking water sources	0.084	0.278	Bedridden due to illness in childhood	0.055	0.228
Childhood household electricity status	0.416	0.493	Adulthood education level	5.788	4.017
Experiencing starvation in childhood	0.324	0.468	Adulthood occupation type	2.817	1.418
Control variables			Adulthood monthly household expenditure (in log)	6.973	1.053
Age	60.845	9.282	Adulthood household registration	0.121	0.326
Gender	0.492	0.500	Adulthood smoking	0.466	0.499
Marital status	0.880	0.325	Adulthood drinking	0.407	0.491
Number of siblings	4.322	2.018	Adulthood physical activity	0.433	0.496

### 4.2 Empirical analysis

**Figure 1:**
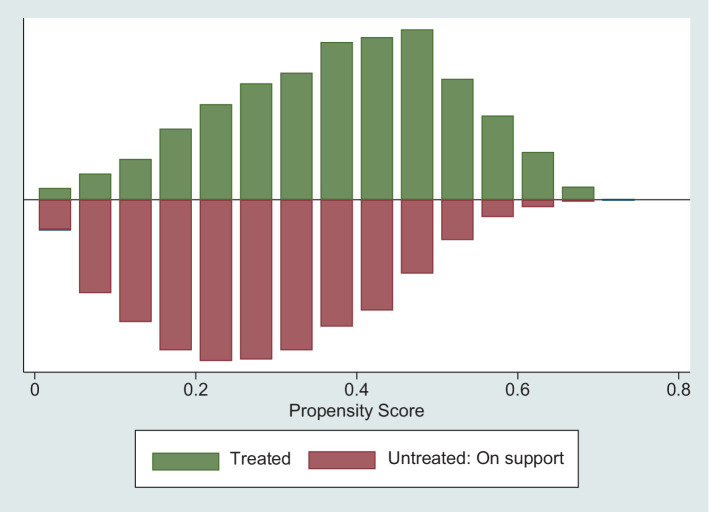
The common support of propensity score.

In this study, we estimated the relationship between childhood SES and the health of middle-aged and older adult individuals by using K-nearest neighbor matching, radius matching and kernel matching methods. According to the common practice in existing literature, we set the K-nearest neighbor matching parameter to 2, the radius matching parameter to 0.01, and the kernel matching parameter to 0.06 (49). To test the matching quality, it is necessary to observe whether the model satisfies the common support assumption. If the matching overlap area between the treatment and control groups is too small, samples outside the overlap area cannot be matched effectively and are removed, which in turn leads to biased estimates. From Figure 1 we can see that most of the sample observations are on common support, indicating that the model satisfies the common support assumption.

Table 2 presents the results of the balance test. The results show that the value of pseudo-R^2^ decreases from 0.081 before matching to 0.001 after matching. The likelihood Ratio (LR) statistic decreased from 999.11 before matching to 10.58 after matching. The joint significance test shows that the significance levels of the covariates have also changed significantly. In addition, both mean bias and median bias of covariates are substantially reduced after matching. Therefore, the propensity score matching reduced the difference in covariates between the treatment and control groups, indicating that the model is well balanced after matching.

**Table 2:** Balance test.

Matching methods	Ps *R*^2^	LR chi^2^	*p* value	Mean bias	Median bias
Before matching	0.081	991.11	0.000	16.4	12.5
K-nearest neighbor matching	0.002	17.69	0.221	1.9	1.5
Radius matching	0.001	8.79	0.844	1.3	1.0
Kernel matching	0.001	5.27	0.982	0.9	0.7
Mean	0.001	10.58	0.682	1.4	1.1

Table 3 describes the results of PSM analysis, which determine the relationship between childhood SES and different dimensions of health in middle and old age. As shown in Model 1, childhood SES has a significant positive correlation with comprehensive health of middle-aged and older adult individuals. Specifically, the high SES group in childhood was on average 5.1% more likely to have good self-reported health in middle and old age than their counterparts from low SES group. The results of Model 1 are significant at the 1% level across various matching methods. In terms of objective and subjective health, Model 2 and 3 also exhibit comparable findings that reveal a negative correlation with chronic illnesses and depression during middle and old age due to high childhood SES. As a result, high SES in childhood is significantly positively correlated with good health in middle and old age, which supported hypothesis 3. It is worth noting that childhood SES has varying relationships with different health dimensions. In terms of the size and significance level of the ATT, the correlation between childhood SES and subjective health is greater than the correlation with objective health.

**Table 3:** The relationship between childhood SES and health in middle and old age.

Matching method	Matching parameters	Comprehensive health	Objective health	Subjective health
		Model 1	Model 2	Model 3
K-nearest neighbor matching	*K* = 2	0.050^***^	−0.022^*^	−0.716^***^
		(0.014)	(0.013)	(0.196)
Common support sample size	/	9,861	9,861	9,861
Radius matching	δ = 0.01	0.049^***^	−0.025^**^	−0.779^***^
		(0.010)	(0.011)	(0.140)
Common support sample size	/	9,861	9,861	9,861
Kernel matching	k: epan bw: 0.06	0.051^***^	−0.025^**^	−0.823^***^
		(0.009)	(0.011)	(0.143)
Common support sample size	/	9,861	9,861	9,861

Significant at * 10%, **5%, ***1%. Standard errors are in parentheses. Standard errors are estimated by bootstrap (with 500 replications).

### 4.3 Robustness check for comprehensive health

In this section, we first perform the robustness check by alternating childhood SES with whether respondents experienced starvation in childhood to perform robustness test. In this study, the respondents experienced childhood at the beginning or before reform and opening up in China. At this time, people’s productivity was more agriculturally dependent because China had not yet undergone rapid urbanization. Thus, experiencing starvation in childhood reflects, to some extent, the original family’s material living conditions. Therefore, the incidence of childhood starvation has a close association with factors such as parents’ education and occupation (50). In addition, to reduce the estimation bias caused by social famines and regional differences in agricultural production, we added whether respondents experienced the 1959–1961 famine or not and the proportion of individuals who experienced starvation in childhood in the same city as control variables in our model. The Model 1 of Table 3 demonstrate that experiencing starvation in childhood has a significant negative association with health in middle and old age, which is consistent with the baseline estimates.

We also perform the robustness check by eliminating the effects of SES changes in childhood. Since the age range of childhood is confined to 0–17 years, change in individual SES during this period may cause bias. Against this backdrop, we exclude samples with changes in childhood SES and re-estimate the relationship between childhood SES and health in middle and old aged. From Model 2 of Table 3 we can see that, after eliminating the effects of SES changes in childhood, the results remain robust.

### 4.4 The associative mediation effects of childhood SES on health in middle and old age

According to theoretical analysis, we identify three mechanisms that explain how childhood SES is associated with the health of middle-aged and older adult individual, they are childhood health status, adulthood SES, and adulthood lifestyles. Table 4 presents the decomposed results of the associative mediation effects. The results of Table 4 show that adulthood SES plays the largest associative mediation effect, followed by childhood health and adulthood lifestyles. Within the adulthood SES groups, education years, occupation type, average monthly household expenditure, and household registration are all statistically significant at the 1% level, this supported hypothesis 2. The percentage of indirect correlation on the total correlation of education years and occupation type are both above 25%. The associative mediation effects of adulthood SES reflects that parents with a high SES increase investment in their children’s education, leading to improved health productivity in adulthood. Furthermore, higher levels of education allow individuals to work in higher-paying occupations, reducing financial constraints related to maintaining and improving health in middle and old age. In addition, self-reported health in childhood and physical activity in adulthood also partially explain the significant positive association of childhood SES with health in later life, which partially supported hypotheses 1 and 3. Overall, the results of mediation analysis suggest that childhood SES has both direct and indirect correlations with health in middle and old age.

**Table 4:** Robustness check.

Matching method	Matching parameters	Self-reported health	Self-reported health
		Model 1	Model 2
K-nearest neighbor matching	*K* = 2	−0.033^***^	0.024
		(0.014)	(0.026)
Common support sample size	/	9,807	5,329
Radius matching	δ = 0.01	−0.037^***^	0.037^**^
		(0.010)	(0.017)
Common support sample size	/	9,807	5,329
Kernel matching	k: epan bw: 0.06	−0.037^***^	0.043^**^
		(0.009)	(0.018)
Common support sample size	/	9,807	5,329

Significant at * 10%, **5%, ***1%. Standard errors are in parentheses. Standard errors are estimated by bootstrap (with 500 replications). Model 1 replaces the childhood SES variable. Model 2 eliminates the effects of SES changes in childhood.

### 4.5 The associative mediation effects of adulthood SES upward mobility on relationship between childhood SES and health in middle and old age

As adulthood SES has a significant associative mediation effect, we investigate the long-term health relationships of adulthood SES upward mobility and validate the social mobility model. Model 1 of Table 6 illustrates that the marginal effect of low childhood SES on health in middle and old age without controlling for adulthood SES upward mobility. In Model 2 of Table 6, we control for the variable of SES upward mobility in adulthood based on Model 1. The marginal effects of Model 1 and Model 2 of Table 6 are all statistically significant at the 1% level. Comparing Model 1 to Model 2 reveals that the association of low childhood SES with poor health in middle and old age increased approximately 1.41 times after controlling for adulthood SES upward mobility. The results suggest that the negative effects of early life hardship are difficult to reverse over the life course. However, this finding does not indicate that individuals are incapable of overcoming childhood SES disadvantages. Through SES upward mobility in adulthood, individuals can partially alleviate the association of childhood SES disadvantage with poor health in middle and old age. Thus, a comprehensive framework including the critical period model, the cumulative risk model and social mobility model is necessary to interpret the mechanism between childhood SES and health in middle and old age (Table 6).

**Table 5:** The associative mediation effects of childhood SES on health in middle and old age.

Variables	Self-reported health		
	Coefficients	SD	Mediating effect %
Childhood health status			
Childhood self-reported health			
Total correlation	0.352^***^	0.073	
Direct correlation	0.334^***^	0.073	
Indirect correlation	0.018^***^	0.007	5.13
Bedridden due to illness in childhood			
Total correlation	0.352^***^	0.072	
Direct correlation	0.350^***^	0.072	
Indirect correlation	0.002	0.003	0.57
Adulthood SES			
Years of education			
Total correlation	0.358^***^	0.072	
Direct correlation	0.213^***^	0.074	
Indirect correlation	0.145^***^	0.019	40.50
Occupation type			
Total correlation	0.387^***^	0.073	
Direct correlation	0.277^***^	0.073	
Indirect correlation	0.110^***^	0.016	28.42
Average monthly household expenditure			
Total correlation	0.354^***^	0.072	
Direct correlation	0.321^***^	0.073	
Indirect correlation	0.033^***^	0.009	9.32
Urban household registration			
Total correlation	0.359^***^	0.072	
Direct correlation	0.315^***^	0.073	
Indirect correlation	0.044^***^	0.015	12.26
Adulthood lifestyle			
Smoking			
Total correlation	0.353^***^	0.072	
Direct correlation	0.352^***^	0.072	
Indirect correlation	0.001	0.002	0.28
Drinking			
Total correlation	0.358^***^	0.072	
Direct correlation	0.352^***^	0.072	
Indirect correlation	0.006	0.006	1.68
Physical activity			
Total correlation	0.353^***^	0.072	
Direct correlation	0.344^***^	0.072	
Indirect correlation	0.009^*^	0.005	2.55

**Table 6:** The associative moderation effects of SES upward mobility in adulthood.

Variables	Model (1)		Model (2)	
	Marginal effect	SD	Marginal effect	SD
Childhood low SES	−0.056^***^	(0.011)	−0.079^***^	(0.012)
Age	−0.003^***^	(0.001)	−0.002^***^	(0.001)
Gender	0.052^***^	(0.011)	0.041^***^	(0.011)
Marital status	0.006	(0.019)	0.004	(0.019)
Number of siblings	−0.007^***^	(0.003)	−0.007^***^	(0.003)
Number of children	−0.020^***^	(0.004)	−0.019^***^	(0.004)
Medical insurance	−0.020	(0.032)	−0.025	(0.031)
Biological mother died prematurely	−0.038^***^	(0.012)	−0.037^***^	(0.012)
Mother was sick in bed for a long-time during respondent’s childhood	−0.064^***^	(0.017)	−0.064^***^	(0.017)
Mother had severe physical disabilities during respondent’s childhood	−0.045	(0.033)	−0.045	(0.033)
Mother was mentally ill during respondents’ childhood	−0.085^**^	(0.040)	−0.078^**^	(0.039)
Biological father died prematurely	0.004	(0.011)	0.005	(0.012)
Father was sick in bed for a long-time during respondent’s childhood	−0.055^***^	(0.021)	−0.054^***^	(0.021)
Father had severe physical disabilities during respondent’s childhood	−0.064^*^	(0.033)	−0.061^*^	(0.033)
Father was mentally ill during respondents’ childhood	0.010	(0.042)	0.010	(0.042)
Adulthood SES upward mobility	/	/	0.071^***^	(0.013)

Significant at * 10%, **5%, ***1%. Standard errors are in parentheses.

### 4.6 Heterogeneity analysis

In this section, we explore the urban–rural heterogeneity of the correlation between childhood SES and health in middle and old age. In view of the urban–rural dual economic structure in China, there exist a significant gap between urban and rural in economic and social development. The correlation between childhood SES and health in middle and old age may exist urban–rural differences. Table 7 presents that childhood SES has a significant positive association with the health of middle-aged and older adult individuals in rural areas. However, for the urban sample, only the estimate derived from the radium matching method is significant. As a result, the correlation between childhood SES and health in middle and old age is more pronounced in rural residents than in their urban counterparts.

**Table 7:** The relationship between childhood SES and health in middle and old age by household registration and gender.

Matching method	Matching parameters	Urban	Rural	Rural male	Rural female
K-nearest neighbor matching	*K* = 2	0.067^*^	0.035^***^	0.017	0.045^**^
		(0.045)	(0.014)	(0.019)	(0.020)
Common support sample size	/	690	9,061	4,464	4,618
Radius matching	δ = 0.01	0.070^*^	0.032^***^	0.032^**^	0.036^**^
		(0.037)	(0.009)	(0.013)	(0.015)
Common support sample size	/	679	9,060	4,464	4,616
Kernel matching	k: epan bw: 0.06	0.047	0.034^***^	0.033^**^	0.038^**^
		(0.033)	(0.010)	(0.013)	(0.015)
Common support sample size	/	690	9,061	4,464	4,618

Significant at * 10%, **5%, ***1%. Standard errors are in parentheses. Standard errors are estimated by bootstrap (with 500 replications).

We also investigate the gender differences in the correlation between childhood SES and health in middle and old age. Since the long-term relationship between childhood SES and health is significant only in rural areas, we limit our sample to rural residents for gender heterogeneity analyses. By comparing the results of rural male and rural female in Table 7, we find that the absolute size and significance level of ATT are higher in females than males. In other words, a woman’s health in middle and old age is more likely to be associated with her childhood SES.

## 5 Discussion

This study aims to explore the long-term relationship between childhood SES and health. The results of this study indicate that high childhood SES has a significant positive association with good health in middle and old age, and these results are robust to different health indices, childhood SES definitions and to eliminating the effect of childhood SES changes. Specifically, groups with high childhood SES have better self-reported health, lower chronic disease prevalence and CESD scores in middle and old age. Decomposition of associative mediation effect shows that adulthood SES, childhood health status, and adulthood healthy behaviors serve as significant mediators in the relationships between childhood SES and health in middle and old age. These findings are consistent with the predictions of the theoretical analysis. Results of the heterogeneity analysis present that the health of middle-aged and older adult individuals in rural areas is more likely to be associated with childhood SES compared to their urban counterparts. At the same time, childhood SES is more closely associated with the health of women in middle and old age than with that of men.

Our findings are contrary to the results of some previous studies from other countries, which have suggested the critical period and accumulation risks models (5, 12, 20, 34). In this study, although the correlation between childhood SES and health persists into middle and old age, upward SES mobility in adulthood is effective in mitigating the correlation between childhood disadvantage and poor health in middle and old age. Therefore, the findings of this study combine the interpretations of the critical Period and social mobility models, and breaks down the cumulative risks model to a degree. The disparities in findings between this study and some previous studies can be attributed to the differences in historical process, social and economic conditions between China and other countries. A possible explanation for this finding might be that China resumed the college entrance examination in 1977, which provided a valuable access to higher education and an opportunity for disadvantaged groups to change their destinies (51, 52). Subsequently, China established a nine-year compulsory education system (including primary and junior secondary education) in 1985. School-aged children have access to 9 years of compulsory education regardless of their gender, ethnicity and household income. As a human capital investment, raising education level contribute to individuals’ SES upward mobility, thereby mitigating the adverse effects of early SES disadvantage.

Another possible explanation for our finding is that dramatic changes in socio-economic structure have generated numerous opportunities for upward mobility for SES disadvantaged groups in China. It is well known that reform and opening-up policy implemented in 1978 has driven high economic development and industrial structure transformation in China. The occupational structure that directly related to social stratification has also changed significantly, characterized by a rapid growth in middle and upper-level occupations. Consequently, occupational diversification provides an important vehicle for SES disadvantaged groups to earn high income and accumulate wealth (53). According to the statistics, the rate of intergenerational upward social mobility has increased by almost 9% since 1980 and continues to rise overtime in China. Under this favorable external environment, individuals take advantage of historical opportunities and show initiative to achieve upward mobility, which effectively reduces the negative association between early SES and health in middle and old age.

Heterogeneity analyses reveal that the correlation between childhood SES and health in middle and old age was more pronounced in rural areas and female samples. The reason can be attributed to the dual economic structure that existed between urban and rural areas during the planned economy era in China. In this period, the Country allocated material resources primarily to urban areas to promote industrialization. Consequently, individuals with an urban household registration can benefit from institutional advantages, and their material living conditions are not only less disparate but also generally better than individuals with rural household registration. It is worth noting that income and wealth were not criteria for social stratification during the planned economy era in China. Instead, individual’s SES was primarily manifested in material living resources. As a result, health disparities in adulthood resulting from variation in childhood SES are more significant in rural areas.

The increased vulnerability of women’s health to childhood SES in middle and old age can be attributed to the traditional concepts of “boy preference” in China. Traditional concepts of “boy preference” is more widespread in rural areas of China, particularly when a family is experiencing socio-economic hardships and has limited resources for survival. In this case, boys usually enjoy more family resources, resulting in discrimination against girls during their childhood and subsequently compromising their health in adulthood (54). “Boy preference” is also reflected in the unequal allocation of education resources. Parents often prioritize investing in their sons’ education, viewing them as future “pillars” of the family. Previous studies also confirm that unequal access to education based on gender contributes to health disparities among middled-aged and older adult individuals (55).

This study contributes to the literature on the long-term relationship between childhood SES and health by providing more evidence from China. In this study, we not only theoretically analyzed the relationship between childhood SES and health in middle and old age, but also employed more objective indices of childhood SES that are suitable for the special realities of the Chinese society. In addition, we also integrated the critical period and social mobility models into a unified framework to analyze the mechanism between childhood SES and health in middle and old age. The results of sub-sample groups reflect the urban–rural dual economic structure and the traditional concepts of “boy preference” in China. The findings of this study suggest that early interventions starting in childhood are necessary for healthy aging. The government should improve the material living conditions of children from disadvantaged families and guarantee their right to education, particularly for girls in remote rural areas. In addition, it is crucial to popularize health knowledge and encourage healthy lifestyles for all age groups.

It is important to bear in mind the possible bias that may exist in this study. Given the relatively large time span between childhood and after midlife, the model may contain confounding factors that are challenging to observe. Under this situation, we included as many covariates as possible in the PSM model based on the existing literature and data. However, some covariates may be occurred after the treatment variables in our model. For example, the educational level of parents may affect their health during the childhood of their children. It is widely accepted that including pre-treatment variables is crucial for causal inference, particularly in cases involving confounding factors. Therefore, the estimates of this study may be biased if viewed from the perspective of causal inferencing. It is worth noting that findings of this study have been demonstrated to be consistent in a variety of robustness tests and heterogeneity analyses, confirming a substantial association between childhood SES and health in middle and old age.

Regarding future research, one interesting direction is to investigate the causal relationship between childhood adversity and adulthood life attitudes and experiences by using large longitudinal tracking data across decades in China.

## Data Availability

The raw data supporting the conclusions of this article will be made available by the authors, without undue reservation.
