# Supplementary material for: The relationship between childhood SES and health in middle and old age: evidence from China
Source: Front Public Health. 2024 Oct 15;12:1396420. doi: 10.3389/fpubh.2024.1396420 (PMC11520600; doi:10.3389/fpubh.2024.1396420)
Supplement: Supplementary file 1 [file Data_Sheet_1.docx]

*Table 1*

sum shealth18 chronic CESD fedu medu moccu foccu childhukou ranliao water dian age gender marriage bsize csize insurance mmd msick mdefor mmind mfd fsick fdefor fmind

*Principal components analysis (PCA)*

pwcorr fedu medu moccu foccu childhukou ranliao water dian,sig star(0.1) //Correlation analysis of variables

factortest fedu medu moccu foccu childhukou ranliao water dian //Kmo and sphericity tests

pca fedu medu moccu foccu childhukou ranliao water dian //Principal components analysis

estat loading,cnorm(eigen) //Loadings analysis of principal components

predict f1 //Principal component analysis (PCA) coefficient

format f1 %9.3f

gen index=f1 //Childhood socio-economic status index

gen index1=1 if index>=-4.93e-10

replace index1=0 if index<-4.93e-10

*Model 1 of Table 3 (K-nearest neighbour matching)*

psmatch2 index1 age gender marriage bsize csize insurance mmd msick mdefor mmind mfd fsick fdefor fmind, outcome(shealth18) neighbor(2) ate ties logit common quietly

bootstrap r(att)r(atu)r(ate),reps(500): psmatch2 index1 age gender marriage bsize csize insurance mmd msick mdefor mmind mfd fsick fdefor fmind, outcome(shealth18) neighbor(2) ate ties logit common quietly

*Table 2 (Balance test for K-nearest neighbour matching)*

pstest age gender marriage bsize csize insurance mmd msick mdefor mmind mfd fsick fdefor fmind, both

*Figure 1*

Psgraph //The common support of propensity score

*Model 1 of Table 3 (Radius matching)*

psmatch2 index1 age gender marriage bsize csize insurance mmd msick mdefor mmind mfd fsick fdefor fmind, outcome(shealth18) radius cal(0.01) ate ties logit common quietly //Radius matching

bootstrap r(att)r(atu)r(ate),reps(500):psmatch2 index1 age gender marriage bsize csize insurance mmd msick mdefor mmind mfd fsick fdefor fmind, outcome(shealth18) radius cal(0.01) ate ties logit common quietly

*Table 2 (Balance test for radius matching)*

pstest age gender marriage bsize csize insurance mmd msick mdefor mmind mfd fsick fdefor fmind, both

*Model 1 of Table 3 (Kernel matching)*

psmatch2 index1 age gender marriage bsize csize insurance mmd msick mdefor mmind mfd fsick fdefor fmind, outcome(shealth18) kernel ate ties logit common quietly

bootstrap r(att)r(atu)r(ate),reps(500):psmatch2 index1 age gender marriage bsize csize insurance mmd msick mdefor mmind mfd fsick fdefor fmind, outcome(shealth18) kernel ate ties logit common quietly

*Table 2 (Balance test for kernel matching)*

pstest age gender marriage bsize csize insurance mmd msick mdefor mmind mfd fsick fdefor fmind, both graph

*Model 2 of Table 3 (K-nearest neighbor matching)*

psmatch2 index1 age gender marriage bsize csize insurance mmd msick mdefor mmind mfd fsick fdefor fmind, outcome(chronic) neighbor(2) ate ties logit common quietly

bootstrap r(att)r(atu)r(ate),reps(500):psmatch2 index1 age gender marriage bsize csize insurance mmd msick mdefor mmind mfd fsick fdefor fmind, outcome(chronic) neighbor(2) ate ties logit common quietly

*Model 2 of Table 3 (Radius matching)*

psmatch2 index1 age gender marriage bsize csize insurance mmd msick mdefor mmind mfd fsick fdefor fmind, outcome(chronic) radius cal(0.01) ate ties logit common quietly

bootstrap r(att)r(atu)r(ate),reps(500):psmatch2 index1 age gender marriage bsize csize mmd msick mdefor mmind mfd fsick fdefor fmind, outcome(chronic) radius cal(0.01) ate ties logit common quietly

*Model 2 of Table 3 (Kernel matching)*

psmatch2 index1 age gender marriage bsize csize insurance mmd msick mdefor mmind mfd fsick fdefor fmind, outcome(chronic) kernel ate ties logit common quietly

bootstrap r(att)r(atu)r(ate),reps(500):psmatch2 index1 age gender marriage bsize csize mmd msick mdefor mmind mfd fsick fdefor fmind, outcome(chronic) kernel ate ties logit common quietly

*Model 3 of Table 3 (K-nearest neighbor matching)*

psmatch2 index1 age gender marriage bsize csize insurance mmd msick mdefor mmind mfd fsick fdefor fmind, outcome(CESD) neighbor(2) ate ties logit common quietly

bootstrap r(att)r(atu)r(ate),reps(500): psmatch2 index1 age gender marriage bsize csize insurance mmd msick mdefor mmind mfd fsick fdefor fmind, outcome(CESD) neighbor(2) ate ties logit common quietly //求标准误

*Model 3 of Table 3 (Radius matching)*

psmatch2 index1 age gender marriage bsize csize insurance mmd msick mdefor mmind mfd fsick fdefor fmind, outcome(CESD) radius cal(0.01) ate ties logit common quietly

bootstrap r(att)r(atu)r(ate),reps(500):psmatch2 index1 age gender marriage bsize csize insurance mmd msick mdefor mmind mfd fsick fdefor fmind, outcome(CESD) radius cal(0.01) ate ties logit common quietly

*Model 3 of Table 3 (Kernel matching)*

psmatch2 index1 age gender marriage bsize csize insurance mmd msick mdefor mmind mfd fsick fdefor fmind, outcome(CESD) kernel ate ties logit common quietly

bootstrap r(att)r(atu)r(ate),reps(500):psmatch2 index1 age gender marriage bsize csize insurance mmd msick mdefor mmind mfd fsick fdefor fmind, outcome(CESD) kernel ate ties logit common quietly

*Model 1 of Table 4 (K-nearest neighbor matching)*

psmatch2 ses age gender marriage bsize csize insurance mmd msick mdefor mmind mfd fsick fdefor fmind famine chungry, outcome(shealth18) neighbor(2) ate ties logit common quietly

bootstrap r(att)r(atu)r(ate),reps(500): psmatch2 ses age gender marriage bsize csize insurance mmd msick mdefor mmind mfd fsick fdefor fmind famine chungry, outcome(shealth18) neighbor(10) ate ties logit common quietly //求标准误

*Model 1 of Table 4 (Radius matching)*

psmatch2 ses age gender marriage bsize csize insurance mmd msick mdefor mmind mfd fsick fdefor fmind famine chungry, outcome(shealth18) radius cal(0.01) ate ties logit common quietly

bootstrap r(att)r(atu)r(ate),reps(500):psmatch2 ses age gender marriage bsize csize insurance mmd msick mdefor mmind mfd fsick fdefor fmind famine chungry, outcome(shealth18) radius cal(0.01) ate ties logit common quietly

*Model 1 of Table 4 (Kernel matching)*

psmatch2 ses age gender marriage bsize csize insurance mmd msick mdefor mmind mfd fsick fdefor fmind famine chungry, outcome(shealth18) kernel ate ties logit common quietly

bootstrap r(att)r(atu)r(ate),reps(500):psmatch2 ses age gender marriage bsize csize insurance mmd msick mdefor mmind mfd fsick fdefor fmind famine chungry, outcome(shealth18) kernel ate ties logit common quietly

*Excluding samples with a change childhood SES*

drop if change==10

drop if rct1==1 | rct1==2

drop if wct==1

drop if dct==1

*Model 2 of Table 4 (K-nearest neighbor matching)*

psmatch2 index1 age gender marriage bsize csize insurance mmd msick mdefor mmind mfd fsick fdefor fmind, outcome(shealth18) neighbor(2) ate ties logit common quietly

bootstrap r(att)r(atu)r(ate),reps(500): psmatch2 index1 age gender marriage bsize csize insurance mmd msick mdefor mmind mfd fsick fdefor fmind, outcome(shealth18) neighbor(2) ate ties logit common quietly

*Model 2 of Table 4 (Radius matching)*

psmatch2 index1 age gender marriage bsize csize insurance mmd msick mdefor mmind mfd fsick fdefor fmind, outcome(shealth18) radius cal(0.01) ate ties logit common quietly

bootstrap r(att)r(atu)r(ate),reps(500):psmatch2 index1 age gender marriage bsize csize insurance mmd msick mdefor mmind mfd fsick fdefor fmind, outcome(shealth18) radius cal(0.01) ate ties logit common quietly

*Model 2 of Table 4 (Kernel matching)*

psmatch2 index1 age gender marriage bsize csize insurance mmd msick mdefor mmind mfd fsick fdefor fmind, outcome(shealth18) kernel ate ties logit common quietly

bootstrap r(att)r(atu)r(ate),reps(500):psmatch2 index1 age gender marriage bsize csize insurance mmd msick mdefor mmind mfd fsick fdefor fmind, outcome(shealth18) kernel ate ties logit common quietly

*Table 5*

khb logit shealth18 index1 || cshealth1, c(age gender marriage bsize csize insurance mmd msick mdefor mmind mfd fsick fdefor fmind)

khb logit shealth18 index1 || cshealth2, c(age gender marriage bsize csize insurance mmd msick mdefor mmind mfd fsick fdefor fmind)

khb logit shealth18 index1 || edu, c(age gender marriage bsize csize insurance mmd msick mdefor mmind mfd fsick fdefor fmind)

khb logit shealth18 index1 || consumption1, c(age gender marriage bsize csize insurance mmd msick mdefor mmind mfd fsick fdefor fmind)

khb logit shealth18 index1 || occupation, c(age gender marriage bsize csize insurance mmd msick mdefor mmind mfd fsick fdefor fmind)

khb logit shealth18 index1 || hukou, c(age gender marriage bsize csize insurance mmd msick mdefor mmind mfd fsick fdefor fmind)

khb logit shealth18 index1 || smoke, c(age gender marriage bsize csize insurance mmd msick mdefor mmind mfd fsick fdefor fmind)

khb logit shealth18 index1 || drink, c(age gender marriage bsize csize insurance mmd msick mdefor mmind mfd fsick fdefor fmind)

khb logit shealth18 index1 || exercise, c(age gender marriage bsize csize insurance mmd msick mdefor mmind mfd fsick fdefor fmind)

*Table 6*

probit shealth18 index3 age gender marriage bsize csize insurance mmd msick mdefor mmind mfd fsick fdefor fmind

probit shealth18 index3 age gender marriage bsize csize insurance mmd msick mdefor mmind mfd fsick fdefor fmind schange1

*Urban/rural subgroups*

keep if childhukou==1

keep if childhukou==0

*Gender subgroups*

keep if gender==1

keep if gender==0

*Column 3 of Table 7*

keep if childhukou==1

*Column 3 of Table 7 (K-nearest neighbor matching)*

psmatch2 ses age gender marriage bsize csize insurance mmd msick mdefor mmind mfd fsick fdefor fmind famine chungry, outcome(shealth18) neighbor(2) ate ties logit common quietly

bootstrap r(att)r(atu)r(ate),reps(500): psmatch2 ses age gender marriage bsize csize insurance mmd msick mdefor mmind mfd fsick fdefor fmind famine chungry, outcome(shealth18) neighbor(2) ate ties logit common quietly //求标准误

*Column 3 of Table 7 (Radius matching)*

psmatch2 ses age gender marriage bsize csize insurance mmd msick mdefor mmind mfd fsick fdefor fmind famine chungry, outcome(shealth18) radius cal(0.01) ate ties logit common quietly

bootstrap r(att)r(atu)r(ate),reps(500):psmatch2 ses age gender marriage bsize csize insurance mmd msick mdefor mmind mfd fsick fdefor fmind famine chungry, outcome(shealth18) radius cal(0.01) ate ties logit common quietly

*Column 3 of Table 7 (Kernel matching)*

psmatch2 ses age gender marriage bsize csize insurance mmd msick mdefor mmind mfd fsick fdefor fmind famine chungry, outcome(shealth18) kernel ate ties logit common quietly

bootstrap r(att)r(atu)r(ate),reps(500):psmatch2 ses age gender marriage bsize csize insurance mmd msick mdefor mmind mfd fsick fdefor fmind famine chungry, outcome(shealth18) kernel ate ties logit common quietly

*Column 4 of Table 7*

keep if childhukou==0

*Column 4 of Table 7 (K-nearest neighbor matching)*

psmatch2 ses age gender marriage bsize csize insurance mmd msick mdefor mmind mfd fsick fdefor fmind famine chungry, outcome(shealth18) neighbor(2) ate ties logit common quietly

bootstrap r(att)r(atu)r(ate),reps(500): psmatch2 ses age gender marriage bsize csize insurance mmd msick mdefor mmind mfd fsick fdefor fmind famine chungry, outcome(shealth18) neighbor(2) ate ties logit common quietly

*Column 4 of Table 7 (Radius matching)*

psmatch2 ses age gender marriage bsize csize insurance mmd msick mdefor mmind mfd fsick fdefor fmind famine chungry, outcome(shealth18) radius cal(0.01) ate ties logit common quietly

bootstrap r(att)r(atu)r(ate),reps(500):psmatch2 ses age gender marriage bsize csize insurance mmd msick mdefor mmind mfd fsick fdefor fmind famine chungry, outcome(shealth18) radius cal(0.01) ate ties logit common quietly

*Column 4 of Table 7 (Kernel matching)*

psmatch2 ses age gender marriage bsize csize insurance mmd msick mdefor mmind mfd fsick fdefor fmind famine chungry, outcome(shealth18) kernel ate ties logit common quietly

bootstrap r(att)r(atu)r(ate),reps(500):psmatch2 ses age gender marriage bsize csize insurance mmd msick mdefor mmind mfd fsick fdefor fmind famine chungry, outcome(shealth18) kernel ate ties logit common quietly

*Column 5 of Table 7*

keep if childhukou==0

keep if gender==1

*Column 5 of Table 7 (K-nearest neighbor matching)*

psmatch2 ses age gender marriage bsize csize insurance mmd msick mdefor mmind mfd fsick fdefor fmind famine chungry, outcome(shealth18) neighbor(2) ate ties logit common quietly

bootstrap r(att)r(atu)r(ate),reps(500): psmatch2 ses age gender marriage bsize csize insurance mmd msick mdefor mmind mfd fsick fdefor fmind famine chungry, outcome(shealth18) neighbor(2) ate ties logit common quietly

*Column 5 of Table 7 (Radius matching)*

psmatch2 ses age gender marriage bsize csize insurance mmd msick mdefor mmind mfd fsick fdefor fmind famine chungry, outcome(shealth18) radius cal(0.01) ate ties logit common quietly

bootstrap r(att)r(atu)r(ate),reps(500):psmatch2 ses age gender marriage bsize csize insurance mmd msick mdefor mmind mfd fsick fdefor fmind famine chungry, outcome(shealth18) radius cal(0.01) ate ties logit common quietly

*Column 5 of Table 7 (Kernel matching)*

psmatch2 ses age gender marriage bsize csize insurance mmd msick mdefor mmind mfd fsick fdefor fmind famine chungry, outcome(shealth18) kernel ate ties logit common quietly

bootstrap r(att)r(atu)r(ate),reps(500):psmatch2 ses age gender marriage bsize csize insurance mmd msick mdefor mmind mfd fsick fdefor fmind famine chungry, outcome(shealth18) kernel ate ties logit common quietly

*Column 6 of Table 7*

keep if childhukou==0

keep if gender==0

*Column 6 of Table 7 (K-nearest neighbor matching)*

psmatch2 ses age gender marriage bsize csize insurance mmd msick mdefor mmind mfd fsick fdefor fmind famine chungry, outcome(shealth18) neighbor(2) ate ties logit common quietly

bootstrap r(att)r(atu)r(ate),reps(500): psmatch2 ses age gender marriage bsize csize insurance mmd msick mdefor mmind mfd fsick fdefor fmind famine chungry, outcome(shealth18) neighbor(2) ate ties logit common quietly

*Column 6 of Table 7 (Radius matching)*

psmatch2 ses age gender marriage bsize csize insurance mmd msick mdefor mmind mfd fsick fdefor fmind famine chungry, outcome(shealth18) radius cal(0.01) ate ties logit common quietly

bootstrap r(att)r(atu)r(ate),reps(500):psmatch2 ses age gender marriage bsize csize insurance mmd msick mdefor mmind mfd fsick fdefor fmind famine chungry, outcome(shealth18) radius cal(0.01) ate ties logit common quietly

*Column 6 of Table 7 (Kernel matching)*

psmatch2 ses age gender marriage bsize csize insurance mmd msick mdefor mmind mfd fsick fdefor fmind famine chungry, outcome(shealth18) kernel ate ties logit common quietly

bootstrap r(att)r(atu)r(ate),reps(500):psmatch2 ses age gender marriage bsize csize insurance mmd msick mdefor mmind mfd fsick fdefor fmind famine chungry, outcome(shealth18) kernel ate ties logit common quietly
